# Effectiveness of an 8-week neck exercise training on pain, jaw function, and oral health-related quality of life in women with chronic temporomandibular disorders: a randomized controlled trial

**DOI:** 10.22514/jofph.2024.005

**Published:** 2024-03-12

**Authors:** Ana Izabela Sobral de Oliveira-Souza, Laís Ribeiro do Valle Sales, Alexandra Daniele de Fontes Coutinho, Daniella Araújo de Oliveira, Susan Armijo-Olivo

**Affiliations:** ^1^Physiotherapy Department, Federal University of Pernambuco, 50670-901 Recife, Pernambuco, Brazil; ^2^Faculty of business and Social Sciences, University of Applied Sciences, 49076 Osnabrück, Germany; ^3^Faculty of Rehabilitation Medicine/Faculty of Medicine and Dentistry, University of Alberta, Edmonton, AB T6G 2G4, Canada

**Keywords:** Temporomandibular joint disorder, Jaw diseases, Neck muscles, Physical therapy modalities, Exercise therapy

## Abstract

To test the effectiveness of an 8-week exercise program targeted to the neck muscles compared to manual therapy, and placebo treatments on orofacial pain intensity, jaw function, oral health-related quality of life (OHRQoL), and jaw range of motion (ROM) in women with Temporomandibular Disorders (TMD). In this randomized controlled trial, fifty-four women (between 18–45 years old) with a diagnosis of myofascial or mixed TMD according to the Research Diagnostic Criteria for TMD (RDC/TMD) were randomized into three groups: Neck motor control training (NTG), Manual Therapy Group (MTG), and Placebo Group (PG). All patients were evaluated with the Visual Analog Scale, Mandibular Function Impairment Questionnaire, Oral Health Impact Profile-14, and jaw Range of Motion (ROM) at baseline, immediately after treatment (after 8 weeks of treatment), one month, and three-month follow-up. For all outcomes, a mixed analysis of variance (ANOVA) with repeated measures was conducted with a Bonferroni *post hoc* test. NTG was significantly better than the PG group on pain and jaw function at the end of treatment, one- and three-month follow-up (Effect Size (ES) >0.7). For OHRQoL, NTG was significantly better than MTG and PG at the end of treatment and at three-month follow-up (ES >0.7). The results of this project are encouraging, and they could be used to guide clinical practice in this field. Exercises targeted to the neck (which require low therapeutic supervision) could be a simple and conservative way to improve pain and disability for women with TMD with neck involvement.

## 1. Introduction

Temporomandibular disorders (TMD) are a group of conditions affecting the stomatognathic system characterized by the presence of preauricular pain, restriction, deviation or noises during jaw movements, and fatigue and pain of the masticatory muscles. In addition, TMD is also commonly associated with other symptoms affecting the head and neck [1, 2]. TMD signs and symptoms occur twice more often in women than in men (2:1) [3, 4, 5, 6], and over 70% of patients with TMD are women. Additionally, women are more severely affected by TMD than men and generally seek treatment more often [7]. TMD is considered an important public health problem, as it is the main source of chronic orofacial pain having a big impact on the quality of life of women with TMD [8, 9]. TMD has been shown to interfere with daily activities, reducing the capacity for work and/or the social interaction of individuals suffering from this condition, and has a big economic impact [10].

Patients with myogenic (characterized by symptoms in the masticatory muscles) or mixed TMD (characterized by the presence of muscular and joint symptoms) present persistent pain, allodynia and hyperalgesia, showing an abnormal function of the central nervous system similar to other chronic pain conditions [11, 12, 13]. They usually present motor dysfunction [13, 14, 15], expressing changes in muscle behavior and function [16, 17]. Specifically, it has been demonstrated that subjects with TMD present lower neck flexor and extensor muscles endurance and force in isometric tasks [18], also an impaired performance of the deep cervical flexor muscles while conducting the craniocervical flexion test (CCFT) [17, 18].

There is abundant evidence showing that neck muscles and structures, and neck functionality are impaired in subjects with TMD [14, 17, 18, 19, 20]. For example, people with jaw pain have worse neck disability and lower values on pressure pain threshold (PPT) of neck muscles than people without jaw pain [21]. In addition, subjects with myogenic TMD diagnoses are more likely to present masticatory and cervical muscle sensitivity, self-reported neck disability, and lower PPT of temporal anterior, sternocleidomastoid (SCOM) and upper trapezius muscles [21].

The association between TMD and neck disorders is thought to occur due to the anatomic proximity and the neurological convergence between the cervical and orofacial regions in the trigeminocervical nucleus [21, 22]. Due to this convergence between orofacial and cervical areas [23], pain in each of the three upper cervical joints and on muscles innervated by the upper cervical spinal nerves could be perceived at any area innervated by the trigeminal nerve. So, impairments in the neuromuscular motor control in the neck could potentially cause an overload of the craniocervical system and consequently lead to pain in related structures such as the orofacial region [18].

Due to the complexity of TMD and also due to their common association with headache, neck pain and disability, the physiotherapeutic approach in clinical settings has been concentrated on improving craniocervical functioning through exercises targeted to the neck [18]. Although none of the treatments applied in any existing trials (splints, exercises, manual therapy, physical agents) can be considered effective for all patients, these treatments are commonly used in clinical practice, and there is evidence that they can be potentially effective for managing patients with TMD based on current literature, although the current evidence is poor. Treatments including neck and head postural exercises and therapeutic exercises to masticatory muscles and/or neck muscles have been shown to be effective to reduce musculoskeletal pain and improving jaw function, force, coordination, endurance, mobility, stability and motor control of the muscle system in these patients [24, 25, 26]. In the same way, manual therapy treatment has been used to improve the range of motion and proprioception, stimulate synovial fluid production and reduce pain in different conditions. In subjects with TMD, manual therapy applied to the neck area has been shown to reduce pain and improve PPT and the range of motion of masticatory and neck muscles [24, 27, 28, 29].

Different neck exercises, especially endurance training of the deep neck muscles, have been used to reduce pain and improve neck motor control in patients with neck pain-related disorders and cervicogenic headache [30, 31, 32]. Several trials have tested the effectiveness of neck motor control in different conditions involving the neck such as neck pain, cervicogenic headache, and whiplash among others, and all of them have shown positive results. However, limited numbers of trials have tested the effectiveness of manual therapy directed to the neck in subjects with TMD.

To our knowledge, none of the previous studies have evaluated in isolation the effectiveness of motor control exercises targeted to neck muscles in improving pain, orofacial function and quality of life in patients with TMD. Therefore, this study provides preliminary and novel evidence regarding the potential use of these exercises for this condition. Thus, the objective of this study was to determine the effectiveness of an 8-week exercise program targeted to the neck muscles compared to manual therapy, and placebo treatments on orofacial pain intensity (main outcome), jaw function, oral health-related quality of life (OHRQoL), and jaw range of motion (ROM) (secondaries outcomes) in patients with TMD immediately after the end of treatment (final evaluation; main time point), at one-month follow-up (four weeks after the end of treatment), and at three-months follow-up (12 weeks after the end of treatment).

It was hypothesized that, after eight weeks of treatment, individuals receiving neck motor control training would significantly reduce their orofacial pain, and would significantly improve their jaw function, OHRQoL and jaw ROM when compared to placebo treatment. It was hypothesized that neck motor control training would have similar results to manual therapy treatment.

## 2. Materials and methods

### 2.1 Study design

This study was a parallel randomized controlled trial (RCT). The assessor (who measured the clinician-assessed outcomes) did not know the group allocation, the therapist did not know the results of the measured data, and the patients were unaware of the study hypothesis (to decrease performance biases). In addition, patients were instructed to not discuss their allocation group with the other participants, to avoid biases. The randomization process was performed by using a website (https://www.randomizer.org/) that provided the random sequence. The randomization sequence was generated by a research assistant not involved in the trial recruitment. To ensure the concealment of allocation, an external researcher, not involved in the study phases, prepared opaque envelopes (sealed and numbered) with the randomization sequence. Envelopes were opened by the therapist after baseline assessment and immediately before starting the treatment. Participants were equally randomized into three groups: Neck Motor Control Training Group (NTG), Manual Therapy Group (MTG), and Placebo Group (PG).

This trial was reported according to the CONSORT guidelines and the Template for Intervention Description and Replication (TIDieR) checklist and guide [33, 34].

### 2.2 Subjects

Participants were recruited from the Department of Dentistry of the University and the community through announcements by advertisements placed in the University’s centers and social media between October 2017 to September 2019. Patients were included based on the following inclusion criteria: women; aged between 18 to 45 years old; orofacial pain for at least six months; and diagnosis of masticatory myofascial pain or mixed TMD (patients who present articular and muscular symptoms in combination) according to the Research Diagnostic Criteria for TMD (RDC/TMD) [35]. Patients were excluded if they had (1) a history of neck or facial trauma; (2) a history of cervical spine and/or craniofacial surgery; (3) a diagnosis of fibromyalgia or rheumatic or neurologic or chronic systemic issues; (4) mental illness; (5) orthodontic treatment ongoing or completed in less than six months; or (6) participants who had been using occlusal splints or regular medication or treated by a physiotherapist for less than six months. An experienced clinician determined the eligibility of the subjects and used the standardized forms from the RDC/TMD to evaluate the patients.

The diagnosis was made with the old RDC/TMD criteria version since the new version of the RDC/TMD criteria was not available in the Portuguese language during the project data collection. The complete standardized RDC/TMD criteria examination was applied to all patients.

#### 2.2.1 Sample size calculation

The Sample size was determined using the G*power software (version 3.1.9.7. Heinrich-Heine-University, Düsseldorf, Germany), based on the primary outcome of this trial, which was orofacial pain intensity (measured by a 0–10 cm Visual Analog Scale (VAS)). This calculation was based on a pilot study conducted by our team. Based on the estimates of the effect size (mean difference) of 2.6 (Standard Deviation (SD) 0.83; ES = 1.4) mm between active groups (NTG and MTG) when compared to PG on VAS, an α = 0.05; and β = 80%, 54 participants were needed in total, being 18 patients per group.

#### 2.2.2 Procedures

Demographic data including age, weight, height, body mass index (BMI), temporomandibular joint (TMJ) pain, difficulty feeding, and headache pain intensity (VAS) were collected for all subjects. In addition, the following outcomes were collected:

### 2.3 Outcome measures

All clinician-assessed outcomes (when the outcomes were not self-reported, *i.e.*, jaw ROM) were collected by two assessors who were blinded to the treatment allocation. The primary outcome measure was self-reported orofacial pain intensity measured with the VAS. Secondary outcomes were self-reported jaw function and oral health-related to quality of life (OHRQoL), and jaw range of motion (ROM) evaluated by a clinician. We also collected neck outcomes; these outcomes will be analyzed and presented in another manuscript that is under preparation. All evaluation procedures and treatments were performed in the Learning and Motor Control Laboratory at the Physiotherapy Department.

#### 2.3.1 Orofacial pain intensity

The orofacial pain intensity was measured by VAS, which is a line with 0 to 10 centimeters, anchored by descriptors at each endpoint. Where, in the left end “0 (zero)” means “no pain”, and in the right end “10” means “worst pain imaginable”. The participants were guided to check a perpendicular line along that axis, at the point that best represented the orofacial pain at rest. The pain intensity was described as the value between the point chosen by the participants to the left endpoint, in cm. The validity and responsiveness [36] of the VAS to measure pain has been recognized and the reliability of VAS has been considered fair to good (Intra-class Correlation Coefficient (ICC): 0.55–0.83) [37, 38, 39].

#### 2.3.2 Jaw function

Jaw function was evaluated by the Mandibular Function Impairment Questionnaire (MFIQ), which permits the classification of the severity of the jaw functional limitation associated with TMD. It is a reliable and valid questionnaire to evaluate jaw function in patients with TMD and has been translated and validated for Brazilian Portuguese [40, 41]. This questionnaire was composed of 17 questions, divided into two dimensions: functional capacity and feeding, with a total score of 68 points. Higher scores represent the worst functional impairment on mandibular function. The smallest detectable difference (SDD) for the total score has been established to be 8 points [41].

#### 2.3.3 Oral health-related quality of life (OHRQoL)

The oral health impact profile—simplified version (OHIP-14) was used to evaluate the OHRQoL. This questionnaire is useful to evaluate the individual functional limitations regard to oral symptoms and emotional and social well-being, showing the impact of oral diseases on the individual’s well-being. The OHIP-14 was developed following a conceptual model of oral health and has seven domains: physical pain, psychological disability, psychological discomfort, functional impairment, physical disability, social disability, and general disability. The questionnaire is composed of 14 questions, and each one can be scored between 0 to 4. The total score is 56 and is calculated by adding all items. The higher the score, the worst the OHRQoL. It is a reliable and valid questionnaire to evaluate the quality of life in patients with TMD and has been translated and validated for Brazilian Portuguese [42, 43].

#### 2.3.4 Jaw range of motion (ROM)

Jaw ROM was evaluated for the following movements: jaw opening without pain, (the participant was instructed to open her mouth as far as possible without pain); right and left lateral excursion (the participant was instructed to move her jaw as far as possible to the right/left side without pain); and protrusion (the participant was instructed to move forward her jaw as far as possible without pain). The jaw ROM was measured with a universal caliper (Digital Universal Caliper, 150 mm, São Paulo-SP, Brazil), according to the RDC/TMD guidelines, which has a good validity to evaluate patients with TMD [35]. The measures were done three times with 30 seconds of interval, and the average between them was used for all analyses.

### 2.4 Intervention

This study had two active treatment groups and one placebo group. As described below.

#### 2.4.1 Neck motor control training group (NTG)

Participants of the NTG performed an 8-week exercises program (specific and progressive) to the flexor and extensors neck muscles as described in the protocol by Falla *et al*. [30, 32]. Patients received instructions and were individually supervised for 30 minutes, once per week for a period of 8 weeks, totaling eight sessions. During each session, the physiotherapist verified the performance of the exercises taught in the previous week and if considered appropriate, exercises were progressed. This program consisted of two phases:

##### 2.4.1.1 Phase 1

The first phase lasted six weeks. Patients performed low-load exercises targeted to the deep neck flexors and extensors neck muscles. This phase is directed to train the deep neck stabilizing muscles (longus colli and longus capitis) [30]. Subjects were instructed to perform a craniocervical flexion movement in a supine relaxed position, aided with visual pressure biofeedback device (Pressure Biofeedback Stabilizer; Chattanooga, Hixson, TN, EUA), placed under the occipital region. The Stabilizer device monitors the cervical lordosis flattening that occurs with the contraction of the longus colli. The exercise started with the biofeedback device inflated initially at 20 mmHg. The participant was required to perform a short craniocervical flexion movement (nodding movement: “yes”), and to maintain it for 10 seconds, with 10 repetitions and 10 seconds of rest between them. This sequence was considered as one series with a duration of 190 seconds in total. Then, the patient was allowed to progress the exercise during five stages of 2 mmHg of increment each, reaching a maximum pressure of 30 mmHg, based on the Stabilizer device. The participant was instructed to do the contraction slowly and smoothly, not allowing retraction or lifting the head from the bed and avoiding the co-contraction of the sternocleidomastoid (SCM) and scalene muscles. The number of repetitions and series were adapted individually, ensuring that the patient did the exercises without pain or discomfort. When possible, subjects started the exercises with a minimum of two series of 10 repetitions, and they could progress until 3 series of 10 repetitions.

In this phase, participants performed also exercises to train the deep extensor neck muscles. They executed movements of cranio-cervical extension, flexion and rotation in a prone position on elbows at 90º, with a neutral neck position. The patients started the exercises with a minimum of one series of 10 repetitions of three seconds each. The goal was to evolve until 3 series of 15 repetitions. When more than one series was done, there was an interval of 2 minutes between the series. The number of repetitions and series were adapted individually, ensuring that they did the exercises without pain or discomfort. The protocol of exercises is available in **Supplementary Figs. 1,2,3,4**.

##### 2.4.1.2 Phase 2

The second phase lasted two weeks and had two strengthening exercises for the neck muscles, using the head weight as a load. The first exercise consisted of strengthening the neck flexor muscles. Subjects were in a supine position, and they were instructed to perform a cranio-cervical flexion followed by a cervical flexion raising the head of the bed. The second exercise consisted of strengthening the cervical extensor muscles. Patients were in a 4-kneeling prone position maintaining the craniocervical region in a neutral position, while they were instructed to do a cervical extension movement. The patients started the exercises with a minimum of one series of 10 repetitions of three seconds each. The goal was to evolve into 3 series of 15 repetitions. When more than one series was done, there was an interval of 2 minutes between the series. The number of repetitions and series were adapted individually, ensuring that the patient did the exercises without pain or discomfort.

##### 2.4.1.3 All phases

During all treatment phases, patients were instructed to perform the same exercises at home one time per day, for eight weeks. The exercises lasted between 15 to 20 minutes per day and should be done without pain or discomfort in the jaw, masticatory muscles or neck. The treatment was done by an experienced physiotherapist in the area of orofacial pain (six years of experience), who did not take part in the recruitment and evaluation phases.

#### 2.4.2 Manual therapy group (MTG)

Subjects assigned to this group received manual therapy once per week for eight weeks, lasting approximately 30 minutes. Each session could be extended for another 10 minutes depending on the patient’s needs. The treatment was done by the same physiotherapist who applied the other treatments.

The following techniques were applied:

Myofascial release to neck muscles (upper trapezius, sternocleidomastoid muscles (SCM), anterior scalene and suboccipital, bilateral for 10 minutes [44, 45].

Postero-anterior (P-A) and lateral glide (I and II levels according to Maitland) [46, 47] of the cervical vertebrae were performed just to relieve pain. The four most painful segments during palpation at the time of the session were mobilized. Three series of ten mobilization movements were performed in each segment.

Also, stretching techniques to the neck muscles were applied by the therapist in the following postures: cervical lateral flexion, cervical flexion, cervical flexion with rotation, and cervical extension, at least for 2 series of 30 seconds to a maximum of 3 series of 30 seconds each. The number of series was decided based on the patient’s capacity to perform it.

The following home activities were recommended for this group:

Relaxing techniques: self-massage with circular movements in the neck muscles and hot pads for 20 minutes every day.

Self-stretching of the neck muscles in cervical lateral flexion, cervical flexion, cervical flexion with rotation, and cervical extension. The number of series was determined by the physiotherapist during the in-clinic visit.

#### 2.4.3 Placebo group (PG)

The patients in this group received a placebo treatment. A therapeutic ultrasound—US (Quark®, Pro Seven 977) machine, which was turned off during the therapy, was used to provide a credible placebo. Subjects were not aware of the placebo intervention. Two minutes of turned-off US were applied to the following muscles: SCM, upper trapezius, and splenius, bilaterally with a one-minute interval between them, one time per week, for eight weeks. The patients in this group did not perform any exercise or stretching at home. The treatment was done by the same physiotherapist who applied the other treatments.

All treatment groups were treated in the Learning and Motor Control Laboratory at the Federal University of Pernambuco, individually by the same experienced physiotherapist in the area of orofacial pain (six years of experience), who did not take part in the recruitment and evaluation phases.

Co-interventions for all groups: Participants were required to refrain from other types of treatments for TMD pain during this treatment phase, including medication. If the patient received another treatment, the patient was oriented to say to the physiotherapist and then this was noted by the researcher. However, in this study, no patient referred that she received other types of treatment.

#### 2.4.4 Study time points

All patients were evaluated immediately at the end of the treatment, four weeks after the end of treatment (one-month follow-up), and 12 weeks after the end of treatment (three-months follow-up).

#### 2.4.5 Compliance with treatment

Participants were treated in the clinic, and they were motivated to perform home exercises (NTG) or follow recommendations (MTG). The compliance with treatment in the clinic was assessed based on attendance at each session. To monitor compliance with exercises/recommendations at home, the patients were asked to perform the exercises/recommendations that they had done at home before the beginning of each session.

#### 2.4.6 Statistical analyses

To test the data distribution the histograms and Kolmogorov-Smirnov test was applied. The primary and secondary outcomes were normally distributed and were described in terms of their means and standard deviations (SD).

To characterize the sample, demographic data and clinical data (*i.e.*, age, body mass index (BMI), orofacial pain, headache pain intensity) were compared between groups by using an ANOVA test and Bonferroni *post-hoc* test. To analyze dichotomous variables (presence of TMJ pain, difficulty to feeding, presence of headache, and presence of neck pain) a chi-square (χ^2^) test was used.

To determine whether there was a difference between groups over time on pain intensity (primary outcome) and jaw function, jaw ROM and OHRQoL (secondary outcomes) a mixed ANOVA with repeated measures was conducted. The within-factor was the time: baseline, final (immediately after the end of treatment), one-month follow-up (four weeks after the end of treatment), and three-month follow-up (12 weeks after the end of treatment), indicating the difference in the same group over time, and the between-factor was treatment (NTG, MTG, PG), indicating the differences between groups over time. A Bonferroni *Post hoc* test was applied after ANOVA to verify where the differences occurred.

All results were performed based on intention-to-treat analysis. In this case, all subjects were analyzed according to the group in which they were allocated, including all dropouts. To impute the missing data from the continuous variables, we use an imputation based on plausible data models, obtained from a distribution specifically designed for each missing data point. The selected imputation method takes a set of predictors and returns a single imputation for each missing entry in the incomplete column. To impute each missing data, all other data in the spreadsheet were consulted. For this, the “MICE” package of the RStudio (2021.09.0 Build 351© 2009-2021 RStudio, PBC) was used [48]. To determine the effect sizes (ES) between groups, Cohen’s *d* index was calculated for all outcomes and interpreted according to Cohen’s guidelines [49]. All data analysis was done with the SPSS (version 29.0.1.0 (171), IBM Innovation Studio) and R software (RStudio 2021.09.0 Build 351© 2009-2021 RStudio, PBC).

## 3. Results

In total 153 volunteers were recruited, however, just fifty-four accomplished the inclusion criteria, and were equally randomized among groups. The flowchart (Fig. 1) presented the sample distribution according to the CONSORT statement and highlights the number and reasons for dropouts. Two patients did not complete the evaluation at the end of the treatment (main time point) and dropped out due to personal reasons (one from the NTG (she had no more transportation to come to our lab) and the other from MTG (she was no longer available to participate)). No differences between groups were identified at baseline in any measures. All demographic data are presented in Table 1.

**Fig. 1.** Study flowchart according to the CONSORT statement. TMD: Temporomandibular Disorders.

**Table 1.** Sample characterization.

Outcomes	NTG Mean (SD) 95% CI	MTG Mean (SD) 95% CI	PG Mean (SD) 95% CI	*p* value	*F*
Age (yr)	26 (6.7) 22.6; 29.3	31.8 (9.8) 26.9; 36.7	28.8 (10.4) 23.6; 33.9	0.170	1.836
BMI (kg/cm)	22.9 (4.4) 20.7; 25.0	23.5 (4.9) 21.0; 25.9	23.3 (4.4) 21.1; 25.4	0.916	0.088
Baseline VAS (0–10 cm)	7.25 (1.7) 6.4; 8.1	6.5 (2.2) 5.4; 7.6	7.0 (1.6) 6.2; 7.8	0.531	0.641
Headache VAS (0–10 cm)	7.6 (1.8) 6.7; 8.6	8.0 (1.9) 6.6; 9.4	8.5 (1.5) 7.5; 8.6	0.381	0.990
	YES/N total (%)	YES/N total (%)	YES/N total (%)	*p* value	χ^2^
Joint pain	17/18 (94.4)	14/18 (77.8)	18/18 (100.0)	0.057	5.731
Mixed TMD diagnosis	12/18 (66.7)	15/18 (83.3)	14/18 (77.8)	0.490	1.418
Myofascial TMD diagnosis	6/18 (33.3)	3/18 (16.7)	4/18 (22.2)	0.490	1.418
Difficulty feeding	11/18 (61.1)	13/18 (77.8)	16/18 (88.9)	0.152	3.765
Headache	15/18 (83.3)	11/18 (61.1)	13/18 (72.2)	0.452	1.587
Neck pain	14/18 (77.8)	13/18 (72.2)	16/18 (88.9)	0.450	1.598

*NTG: neck training group; MTG: manual therapy group; CG: control group: 95% CI: confidence interval; p < 0.005; F: ANOVA results; χ^2^: Chi-square test; NTG: Neck motor control training; PG: Placebo group; SD: standard deviations; BMI: body mass index; VAS: Visual Analog Scale; TMD: Temporomandibular Disorders.*

None of the participants received any co-intervention and did not report any type of adverse event. On average the patients attended 95.8%, 88.2% and 91% of the sessions, in the NTG, MTG and PG respectively.

To determine the consistency of results, two different statistical analyses were run; the intention-to-treat (ITT) analyses with the imputation of missing data (*i.e.*, all people randomized (including people who dropped out) and analyzed in the randomized groups) and per-protocol analyses (*i.e.*, people who complied with the treatment (attended sessions) and stayed in the trial). No significant differences were found between these analyses; therefore, we decided to report the ITT analysis. The results of the per-protocol analysis are available in **Supplementary Tables 1,2,3,4**. As treated analysis (*i.e.*, to determine effect estimates based on treatment received) was not necessary since no participant switched groups; so, everyone was treated in the way they were supposed to be treated.

### 3.1 Primary outcome

All groups significantly improved orofacial pain intensity over time (**Supplementary Table 3**). Also, there were significant differences between NTG and PG groups at the end of the treatment (ES 0.9 (95% CI = 0.2; 1.6)), one-month follow-up (ES 0.8 (95% CI = 0.1; 1.5)) and three-months follow-up (ES 0.7 (95% CI = 0.1; 1.4)), favoring the intervention group (Table 2). No significant differences were observed between NTG and MTG, and MTG and PG at any time point.

**Table 2.** Mean differences between groups of Pain intensity, Jaw function and Oral health-related quality of life.

*Post hoc*Between-group (MD 95% CI) (ES 95% CI)						
Outcomes	Comparison		Baseline	End of Treatment	One-month follow-up	Three-months follow-up
Pain intensity VAS (0–10 cm)	NTG *vs.* MTG	MD (95% CI)	0.6 (−0.7; 1.9)	−0.8 (−2.5; 0.9)	−1.1 (−2.5; 0.3)	−1.0 (−2.3; 0.3)
		ES (95% CI)	0.3 (−1.0; 0.3)	0.3 (−0.4; 1.0)	0.5 (−0.1; 1.2)	0.5 (−0.1; 1.2)
	NTG *vs.* PG	MD (95% CI)	0.1 (−1.0; 1.2)	−1.9 (−3.4; −0.4)*	−1.4 (−2.6; 0.3)*	−1.4 (−2.6; −0.2)*
		ES (95% CI)	0.1 (−0.7; 0.6)	0.9 (0.2; 1.6)	0.8 (0.1; 1.5)	0.7 (0.1; 1.4)
	MTG *vs.* PG	MD (95% CI)	−0.5 (−1.8; 0.8)	−1.1 (−2.9; 0.7)	−0.3 (−1.9; 1.3)	−0.4 (−1.9; 1.1)
		ES (95% CI)	0.3 (−0.4; 0.9)	0.4 (−0.2; 1.1)	0.1 (−0.5; 0.8)	0.1 (−0.6; 0.7)
OHRQoL (0–56 points)	NTG *vs.* MTG	MD (95% CI)	−6.1 (−13.8; 1.6)	−8.5 (−14.5; −2.5)*	−8.3 (−16.3; −0.3)*	−11.7 (−20.9; −2.5)*
		ES (95% CI)	0.5 (−0.1; 1.2)	0.9 (0.3; 1.6)*	0.7 (0.1; 1.4)*	0.9 (0.2; 1.5)*
	NTG *vs.* PG	MD (95% CI)	−0.8 (−5.4; 4.2)	−9.2 (−15.4; −3.0)*	−7.2 (−14.8; 0.4)	−7.3 (−14.4; −0.2)*
		ES (95% CI)	0.1 (−0.5; 0.8)	1.0 (0.3; 1.7)*	0.6 (−0.1; 1.3)	0.7 (0.1; 1.4)*
	MTG *vs.* PG	MD (95% CI)	5.3 (−3.2; 13.8)	−0.1 (−8.2; 6.8)	1.1 (−8.7; 10.9)	4.4 (−5.5, 14.3)
		ES (95% CI)	−0.4 (−1.1; 0.2)	0.1 (−0.6; 0.7)	−0.1 (−0.7; 0.6)	−0.3 (−1.0; 0.3)
Jaw function (score)	NTG *vs.* MTG	MD (95% CI)	−5.4 (−12.8; 2.0)	−5.7 (−12.7; 1.3)	−6.9 (−14.8; 1.1)	−12.5 (−20.1; −4.1)*
		ES (95% CI)	0.5 (−0.2; 1.1)	0.5 (−0.1; 1.2)	0.6 (−0.1; 1.2)	1.0 (0.3; 1.7)
	NTG *vs.* PG	MD (95% CI)	−5.3 (−12.2; 1.6)	−9.5 (−16.3; −2.7)*	−8.0 (−15.0; −1.0)*	−9. (−16.0; −2.0)*
		ES (95% CI)	0.5 (−0.1; 1.2)	0.9 (0.3; 1.6)*	0.8 (0.1; 1.4)*	0.9 (0.2; 1.6)*
	MTG *vs.* PG	MD (95% CI)	0.1 (−7.9; 8.1)	−3.8 (−12.1; 4.5)	−1.1 (−10.2; 8.0)	3.5 (−5.8; 12.8)
		ES (95% CI)	0.0 (−0.6; 0.6)	0.3 (−0.3; 1.0)	0.1 (−0.6; 0.7)	−0.2 (−0.9; 0.4)

NTG: Cervical training group; MTG: Manual therapy group; PG: placebo group; OHRQoL: oral health-related quality of life; MD: Mean difference; ES: standardized effect sizes; CI: confidence interval. *p < 0.05 (statistical significance).

### 3.2 Secondaries outcomes

#### 3.2.1 Jaw function

Jaw function showed an improvement in the NTG (within-group) just at one- and three-months follow-up. No significant differences were observed over time in jaw function for the MTG and PG (**Supplementary Table 3**). Regards to between-group analyses, significant differences occurred between NTG and PG at the end of treatment (ES 0.9 (95% CI = 0.3; 1.6)), one-month follow-up (ES 0.8 (95% CI = 0.1; 1.4)) and three-months follow-up (ES 0.9 (95% CI = 0.2; 1.6)), favoring the intervention group. And between NTG and MTG after three-months follow-up (ES 1 (95% CI = 0.3; 1.7)), favoring the NTG group (Table 2). No difference was verified between MTG and PG.

#### 3.2.2 OHRQoL

The OHRQoL improved at all time points related to the baseline measure for NTG and MTG, but not for PG. However, all groups improved their quality of life during the three-months follow-up (**Supplementary Table 3**). Between-group analyses showed that the NTG was significantly better than MTG and PG at the end of treatment, one-month and three-months follow-up, with a large ES (>0.7), favoring the NTG (Table 2).

#### 3.2.3 Jaw ROM

Jaw ROM did not show a clear improvement after treatments. The NTG improved protrusion and the MTG improved left lateral protrusion at one- and three-months follow-up (within-group analysis) (**Supplementary Table 4**). Between-group analyses showed that the PG obtained significantly better results in protrusion movement than MTG (ES −0.8 (95% CI = −1.4; −0.1)) (Table 3).

**Table 3.** Mean differences between groups on jaw range of motion (ROM).

*Post hoc*Between-group (MD 95% CI) (ES 95% CI)						
Outcomes	Comparison		Baseline	End of Treatment	One-month follow-up	Three-months follow-up
Jaw opening (mm)	NTG *vs.* MTG	MD (95% CI)	−0.9 (−10.9; 7.2)	−2.4 (−8.4, 3.6)	−1.1 (−7.8; 5.6)	0.0 (−5.8; 5.8)
		ES (95% CI)	−0.1 (−0.7; 0.6)	0.3 (−0.4; 0.9)	0.1 (−0.5; 0.8)	0 (−0.6; 0.6)
	NTG *vs.* PG	MD (95% CI)	0.2 (−6.4; 6.8)	3.4 (−2.9; 9.7)	0.3 (−6.6; 7.2)	1.2 (−4.9; 7.3)
		ES (95% CI)	−0.1 (−0.8; 0.5)	−0.4 (−1.0; 0.3)	0.1 (−0.6; 0.7)	−0.1 (−0.8; 0.5)
	MTG *vs.* PG	MD (95% CI)	1.1 (−5.5; 7.9)	5.8 (−0.4; 12.0)	1.4 (−4.5; 7.3)	1.2 (−3.8; 6.2)
		ES (95% CI)	−0.1 (−0.8; 0.5)	−0.6 (−1.3; 0.1)	−0.1 (−0.7; 0.6)	−0.2 (−0.8; 0.5)
Right lateral excursion (mm)	NTG *vs.* MTG	MD (95% CI)	0.4 (−1.2; 2.0)	−0.1 (−1.4; 1.2)	0.5 (−1.0; 2.0)	0.6 (−0.5; 1.7)
		ES (95% CI)	−0.2 (−0.8; 0.5)	0.05 (−0.6; 0.7)	−0.2 (−0.9; 0.4)	−0.4 (−1.0; 0.3)
	NTG *vs.* PG	MD (95% CI)	0.3 (−1.3; 1.9)	−1.2 (−2.8; 0.2)	0.8 (−0.7; 2.3)	0.4 (−0.7; 1.5)
		ES (95% CI)	−0.1 (−0.8; 0.5)	0.6 (−0.1; 1.2)	−0.3 (−1.0; 0.3)	−0.2 (−0.9; 0.4)
	MTG *vs.* PG	MD (95% CI)	−0.1 (−2.0; 1.8)	−1.2 (−2.4; 0.01)	0.3 (−1.0; 1.6)	−0.2 (−1.3; 0.9)
		ES (95% CI)	0.0 (−0.6; 0.7)	0.6 (−0.01; 1.3)	−0.1 (−0.8; 0.5)	0.1 (−0.5; 0.8)
Left lateral excursion (mm)	NTG *vs.* MTG	MD (95% CI)	0.5 (−0.9; 1.9)	0.1 (−1.6; 1.8)	−1.0 (−2.3; 0.3)	0.5 (−0.6; 1.6)
		ES (95% CI)	−0.2; (−0.9; 0.4)	−0.1 (−0.7; 0.6)	0.5 (−0.1; 1.2)	−0.3 (−1.0; 0.3)
	NTG *vs.* PG	MD (95% CI)	0.1 (−1.6; 1.8)	−1.4 (−3.2; 0.4)	−0.8 (−2.2; 0.6)	0.1 (−0.5; 0.7)
		ES (95% CI)	−0.1 (−0.7; 0.6)	0.5 (−0.1; 1.2)	0.4 (−0.3; 1.0)	0.7 (−0.3; 1.7)
	MTG *vs.* PG	MD (95% CI)	−0.4 (−2.1; 1.3)	−1.5 (−3.1; 0.1)	0.2 (−1.4; 1.8)	−0.7 (−1.8; 0.4)
		ES (95% CI)	0.1 (−0.5; 0.8)	0.6 (−0.1; 1.3)	−0.1 (−0.7; 0.6)	0.4 (−0.2; 1.1)
Protrusion (mm)	NTG *vs.* MTG	MD (95% CI)	−0.3 (−1.4; 0.8)	−0.4 (−1.5; 0.7)	−0.1 (−1.5; 0.9)	−0.5 (−1.8; 0.8)
		ES (95% CI)	0.2 (−0.5; 0.8)	0.2 (−0.4; 0.9)	0.1 (−0.6; 0.7)	0.3 (−0.4; 0.9)
	NTG *vs.* PG	MD (95% CI)	0.0 (−1.0; 1.0)	0.9 (−0.1; 1.9)	0.0 (−1.0; 1.0)	0.5 (−0.4; 1.4)
		ES (95% CI)	0.0 (−0.6; 0.6)	−0.6 (−1.3; 0.1)	−0.0 (−0.6; 0.6)	−0.3 (−1.0; 0.3)
	MTG *vs. *PG	MD (95% CI)	0.3 (−0.8; 1.4)	1.3 (0.1; 2.4)*	0.1 (−1.1; 1.3)	1.0 (−0.1; 2.1)
		ES (95% CI)	−0.2 (−0.8; 0.4)	−0.8 (−1.4; −0.1)*	−0.1 (−0.7; 0.6)	−0.6 (−1.3; 0.1)

NTG: Cervical training group; MTG: Manual therapy group; PG: control group; MD: Mean difference; ES: standardized effect size; CI: confidence interval. *p < 0.05 (statistical significance).

## 4. Discussion

The main results from this RCT were that an exercise program targeting neck motor control training for 8 weeks was effective to improve pain, jaw function and OHRQoL, but not jaw ROM in women with TMD immediately after the end of the treatment. Significant improvement was observed in orofacial pain and OHRQoL over time in all treatment groups. Neck motor control training was similar to manual therapy and better than a placebo to improve pain and jaw function. Nevertheless, neck motor control training was better than these treatments to improve OHRQoL. Thus, the hypothesis of the study was therefore partially confirmed.

### 4.1 Orofacial pain

The neck motor control training proposed in this study was able to significantly reduce orofacial pain intensity in patients with TMD. Similar results were obtained in a previous study using neck motor control exercises plus upper neck manual therapy [28]. The orofacial pain relief after exercises targeted to the neck might have occurred due to the neuroanatomical connections between these areas, since the stimulation of the inhibitory downward path through the neck may reduce pain in the trigeminal area [28, 50, 51]. In addition, it is known that low-intensity exercise such as motor control exercise can provide an adequate stimulus to produce exercise-induced hypoalgesia effect in patients with chronic pain [52].

Patients with chronic musculoskeletal (MSK) pain such as patients with TMD have altered sensory input that affects sensorimotor organization and processes within the central nervous system [53]. Specific exercises such as motor-skill training could stimulate cortical neuroplasticity and the organization of altered motor function seen in these patients [53, 54]. Motor control training also could improve task performance and representation of the trained musculature in the primary motor cortex better than general exercises and this could help with brain neuroplasticity and pain modulation [53, 54].

In the present study, pain intensity also improved in the placebo group, similar to the Barbosa *et al*. [55], (2019)’s study, which applied exercise directly to the jaw and showed a progressive decrease in perceived pain for both treatment and placebo groups (simulated laser therapy). The improvement in pain intensity in the placebo treatment may be derived from the participant’s perception and experience of receiving a treatment to reduce pain, added to memories of previous experiences and current expectations [55]. Placebo effects result from the positive psychosocial context, which can influence the patient’s brain, and it is created by treatment expectations; in this way, it should be considered a powerful component in the clinical approach. It is established that the practitioner’s attitudes and competence may influence the magnitude of the placebo effects, which could represent around 37% to 70% of the magnitude of pain relief [56, 57, 58]. Moreover, the patient-therapist alliance contributes to placebo effects and health outcomes and might have contributed to the positive effect seen in these patients [56, 57]. However, we did not formally evaluate expectations and the therapeutic alliance in our study. Future studies should take into consideration these factors.

### 4.2 Jaw function

Jaw function was better in the neck exercise training group compared to a placebo treatment, similar to Barbosa *et al*. [55]. This improvement in jaw function only in the exercise group, different from the pain assessment, and could be explained because the placebo effect is less likely to influence physical impairments but could potentially affect the illness as a subjective perception of the patient experience [57].

Jaw function was not significantly different after treatment between neck motor control training and manual therapy group and both groups improved jaw function similarly. The improvement in jaw function due to the treatment is expected as a consequence of pain reduction, especially in patients with severe functional limitations. In addition, as mentioned previously, jaw disability has been highly associated with neck disability and thus improvement in the neck could be reflected in improvements in jaw function [18]. Based on the close relationship between jaw muscles and neck muscles, it is important to highlight that the majority of patients in this study presented a neck component involved in their clinical condition, such as neck pain. This aspect could also influence the responses observed in the three groups.

### 4.3 OHRQoL

The OHRQoL improved in all treatment groups, but participants receiving neck motor control training improved more significantly than the other groups. It is well known that OHRQoL is related to worse jaw function and orofacial pain and thus the improvement in quality of life could be related to the improvements of these outcomes and facilitate daily professional and/or social activities [8, 59]. The improvement of quality of life observed in all groups could be due to other aspects related to the quality of life such as pain beliefs, mood, ability to cope, and cognitive-emotional aspects among others. In addition, this outcome could be influenced by the patient-therapist relationship, characteristics of the treatment, and the overall healthcare setting, which are relevant contextual factors that could affect treatment outcomes [9, 56, 57]. However, the improvement was more evident in the neck motor control training group, and this aspect could be related to the nature of the treatment, where the patients have a big responsibility for their treatment, consequently influencing directly their self-care.

### 4.4 Jaw ROM

The improvement in the jaw ROM was not clearly observed for any of the groups. This result is in accordance with another study looking at the effects of an intervention protocol directed to the neck region in patients with TMD [28]. It is expected that treatments that target both regions (neck and jaw) could be more effective to improve jaw ROM and function in the masticatory muscles. Future studies should analyze isolated and combined therapies and determine their effectiveness.

### 4.5 Strength and Clinical Implications

To our knowledge, this is the first study that tested a specific neck motor control exercise protocol alone in a group of patients with TMD. This study showed that exercises directed to the neck (which requires low therapeutic supervision) could be useful in the management of patients with chronic jaw pain. This study chose to apply the protocol of exercises alone, to clarify the effectiveness of this type of treatment in isolation and to ensure the internal validity of the study. This study followed the methodological standards of RCTs using good randomization and allocation concealment processes and procedures to decrease performance and contamination biases.

Although the therapist could not be blinded (due to the nature of the therapies), she was blinded to the outcome measures. In addition, patients were blinded to the hypotheses of the study decreasing the possibility of performance biases. Assessors were blinded to the allocation group, which avoids detection biases. Co-interventions were controlled in order to avoid contamination bias. Furthermore, the intention to treat analysis with multiple data imputations was used, since few dropouts were found at the end of treatment, and a higher number was verified at the three-month follow-up evaluation. This analysis strategies improve the statistical power of the data, especially because a multiple imputation method was applied [60]. This type of imputation is more advantageous than the single imputation because it uses several complete data sets and provides both the between- and within-imputation variability. The multiple imputation method provides values that could estimate the variance and the interval of the parameter of interest and has been found to provide unbiased estimates [61, 62].

### 4.6 Limitations of the study

The main limitations of the present study are regarding patient recruitment. The patients were recruited in two distinct ways (specialized health services and advertisements) and by convenience; this may be considered a source of selection bias that could affect the external validity of the study. Although we included two different types of TMD, and this could be interpreted as a confounder, we just included both types of TMD (myogenous and mixed TMD) in the study, since both types have a muscular component, which is the main component to be addressed by the treatment in the present study; yet we ensured that the distribution of the different types of TMD was not different between the groups. Also, it was not possible to blind the therapist to the treatments due to their nature and because the same therapist was involved in the treatment of all groups. It is important to highlight that our sample consisted of adult women, and thus our results might not be generalizable to other populations (*e.g.*, men, older adults and children). An important weakness of this study that should be acknowledged is that in order to avoid a heterogeneous sample and avoid confounding, subjects with several comorbidities such as diagnosis of fibromyalgia, rheumatic, neurologic, or chronic systemic issues were excluded. Based on the characteristics of patients with TMD, is very likely that several patients were excluded, and thus our results apply only to those without these comorbidities.

### 4.7 Future directions

Further studies should explore the effectiveness of neck exercises in a longer period (more than 8 weeks of exercises) alone and combined with exercise for the jaw region and using longer follow-ups (six months or more). Also, studies should include men, a different age range, and different degrees of jaw disability and chronicity to determine whether this protocol would be beneficial for different subgroups.

## 5. Conclusions

Neck motor control training was effective in improving pain intensity, jaw function, and oral health-related quality of life, but not jaw ROM in women with TMD. In addition, exercises targeted to the neck were significantly better than placebo to improve pain, jaw function, and oral health-related quality of life, and significantly better than manual therapy to improve oral health-related quality of life. The results of this project are encouraging, and they could be used to guide clinical practice in this field. Exercises targeted to the neck (which require low therapeutic supervision) could be a simple and conservative way to improve pain and disability for women with TMD with neck involvement.

## 6. Clinical implications

(1) Neck motor control training is effective in improving orofacial pain.

(2) Neck motor control training is better than manual therapy to improve the quality of life in patients with TMD.

(3) Neck motor control training is better than placebo therapy to improve orofacial symptoms.

(4) Neck motor control training and manual therapy applied directly to the neck muscles are good options to treat patients with TMD.

## Supplementary Material


