# Supplementary material for: Effectiveness of an 8-week neck exercise training on pain, jaw function, and oral health-related quality of life in women with chronic temporomandibular disorders: a randomized controlled trial
Source: J Oral Facial Pain Headache. 2024 Mar 12;38(1):40–51. doi: 10.22514/jofph.2024.005 (PMC11773986; doi:10.22514/jofph.2024.005)
Supplement: Supplementary file 1 [file Supplementary-material.docx]

Supplementary material


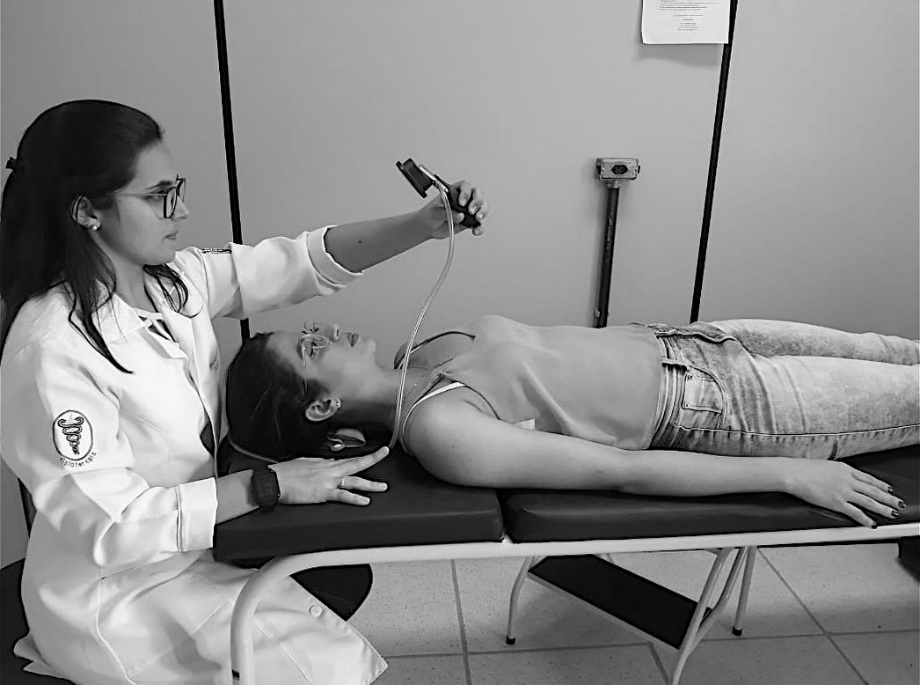


Supplementary Fig. 1. Deep neck flexors training.


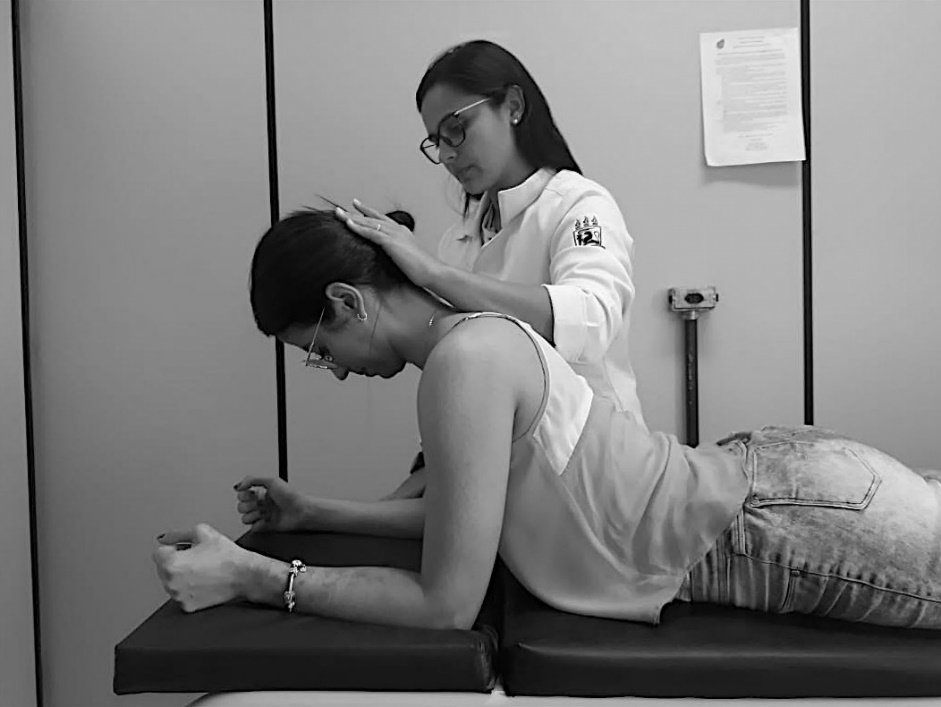


Supplementary Fig. 2. Deep neck extensors training.


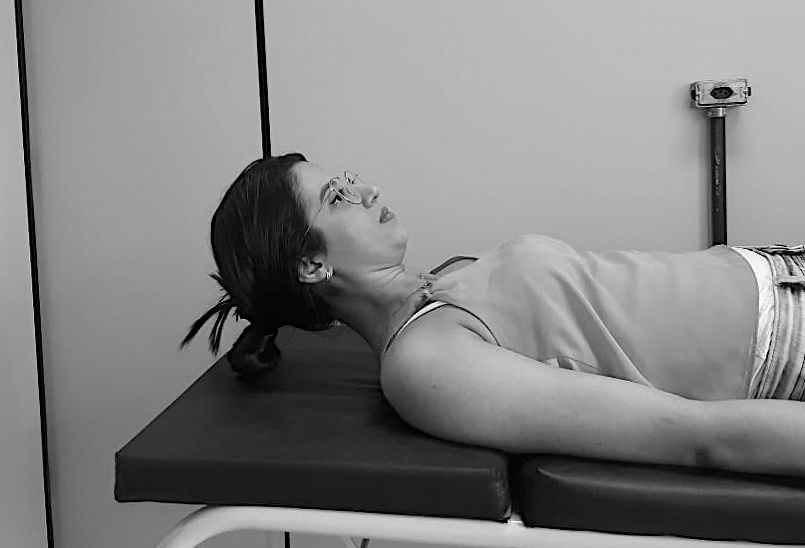


Supplementary Fig. 3. Deep and superficial neck flexors training.


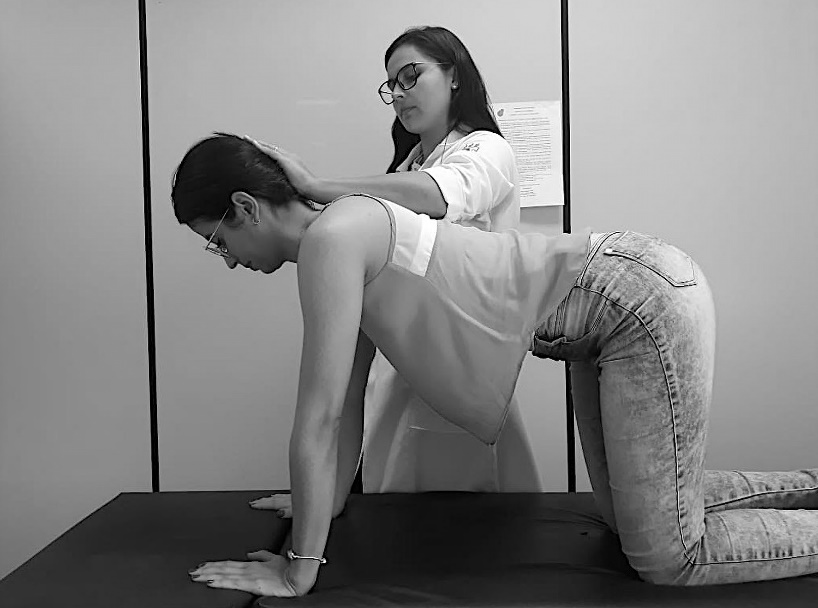


Supplementary Fig. 4. Deep and superficial neck extensors training.

Supplementary Table 1. Mean differences between groups of Pain intensity, Jaw function, and Oral health-related quality of life (PER PROTOCOL).

| *Post hoc*  Between-group (MD CI 95%) (ES CI 95%) | | | | | | |
| --- | --- | --- | --- | --- | --- | --- |
| Outcomes | Comparison |  | Baseline | End of Treatment | One-month  follow-up | Three-months  follow-up |
| Pain | NTG *vs.* MTG | MD (95% CI) | 0.6 (−0.7; 1.9) | −0.9 (−2.9; 1.1) | −1.0 (−2.8; 0.8) | −0.9 (−2.9; 1.2) |
|  |  | ES (95% CI) | 0.3 (−1; 0.3) | 0.4 (−0.3; 1.0) | 0.5 (−0.2; 1.2) | 0.4 (−0.4; 1.2) |
|  | NTG *vs.* CG | MD (95% CI) | 0.1 (−1.0; 1.2) | −2.0 (−4.0; 0.1) | −1.2 (−3.0; 0.5) | −1.6 (−3.6; 0.4) |
|  |  | ES (95% CI) | 0.1 (−0.7; 0.6) | 0.9 (0.2; 1.6) | 0.7 (−0.1; 1.4) | 0.9 (0.1; 1.7) |
|  | MTG *vs.* CG | MD (95% CI) | −0.5 (−1.8; 0.8) | −1.1 (−3.1; 1.0) | −0.2 (−2.0; 1.5) | −0.7 (−2.7; 1.3) |
|  |  | ES (95% CI) | 0.3 (−0.4; 0.9) | 0.4 (−0.3; 1.0) | 0.1 (−0.6; 0.8) | 0.3 (−0.5; 1.1) |
| OHRQoL | NTG *vs.* MTG | MD (95% CI) | −6.1 (−13.8; 1.6) | −8.3 (−16.4; −0.2)* | −8.9 (−19.1; 1.4) | −14.5 (−26.5; −2.3)* |
|  |  | ES (95% CI) | 0.5 (−0.1; 1.2) | 0.9 (0.2; 1.6)* | 0.7 (0.0; 1.4)* | 1.2 (0.4; 2.1)* |
|  | NTG *vs.* CG | MD (95% CI) | −0.8 (−5.4; 4.2) | −9.3 (−17.6; −1.1)* | −4.7 (−15; 5.5) | −11.3 (−22.6; −0.1)* |
|  |  | ES (95% CI) | 0.1 (−0.5; 0.8) | 1 (0.3; 1.7)* | 0.5 (−0.2; 1.2) | 1.2 (0.4; 2)* |
|  | MTG *vs.* CG | MD (95% CI) | 5.3 (−3.2; 13.8) | −1.0 (−9.1; 7.0) | 4.1 (−6.1; 14.4) | 3.2 (−8.6, 15) |
|  |  | ES (95% CI) | −0.4 (−1.1; 0.2) | 0.1 (−0.6; 0.8) | −0.3 (−1.0; 0.4) | −0.2 (−1.0; 0.5) |
| Jaw function | NTG *vs.* MTG | MD (95% CI) | −5.4 (−12.8; 2.0) | −5.7 (−14.8; 3.3) | −5.4 (−15.1; 4.3) | −14.4 (−27.2; −1.7)* |
|  |  | ES (95% CI) | 0.5 (−0.2; 1.1) | 0.5 (−0.1; 1.2) | 0.5 (−0.2; 1.2) | 1.1 (0.3; 2.0)* |
|  | NTG *vs.* CG | MD (95% CI) | −5.3 (−12.2; 1.6) | −9.3 (−18.5; −0.2)* | −7.5 (−17.2; 2.2) | −10.6 (−22.8; 1.7) |
|  |  | ES (95% CI) | 0.5 (−0.1; 1.2) | 0.9 (0.2; 1.6)* | 0.7 (0.1; 1.5)* | 1 (0.2; 1.8) |
|  | MTG *vs.* CG | MD (95% CI) | 0.1 (−7.9; 8.1) | −3.6 (−12.7; 5.4) | −2.1 (−11.8; 7.6) | 3.8 (−8.7; 16.4) |
|  |  | ES (95% CI) | 0 (−0.6; 0.6) | 0.3 (−0.4; 1.0) | 0.2 (−0.5; 0.9) | −0.2 (−1.0; 0.5) |

NTG: Cervical training group; MTG: Manual therapy group; CG: control group; OHRQoL: oral health-related quality of life; CI: confidence interval; MD: Mean difference; ES: standardized effect sizes. **p* < 0.05.

Supplementary Table 2. Mean differences between groups of Jaw range of motion (PER PROTOCOL).

| Outcomes | Comparison |  | Baseline | End of treatment | One-month follow-up | Three-months follow-up |
| --- | --- | --- | --- | --- | --- | --- |
| Jaw opening | NTG *vs.* MTG | MD (95% CI) | −0.9 (−10.9; 7.2) | −2.3 (−10.0; 5.2) | −1.5 (−9.3; 6.2) | 0.9 (−7.7; 9.6) |
|  |  | ES (95% CI) | −0.1 (−0.7; 0.6) | 0.3 (−0.4; 0.9) | 0.2 (−0.5; 0.9) | −0.1 (−0.9; 0.7) |
|  | NTG *vs.* CG | MD (95% CI) | 0.2 (−6.4; 6.8) | 3.5 (−4.2; 11.2) | −1.4 (−9.1; 6.3) | 1.2 (−7.1; 9.5) |
|  |  | ES (95% CI) | −0.1 (−0.8; 0.5) | −0.4 (−1.0; 0.3) | 0.1 (−0.5; 0.8) | −0.1 (−0.9; 0.6) |
|  | MTG *vs.* CG | MD (95% CI) | 1.1 (−5.5; 7.9) | 5.9 (−1.7; 13.5) | 0.1 (−7.6; 7.9) | 0.3 (−8.2; 8.8) |
|  |  | ES (95% CI) | −0.1 (−0.8; 0.5) | −0.6 (−1.3; 0.1) | −0.01 (−0.7; 0.7) | −0.03 (−0.8; 0.7) |
| Right lateral excursion | NTG *vs.* MTG | MD (95% CI) | 0.4 (−1.2; 2.0) | −0.4 (−2.0; 1.3) | 0.5 (−1.3; 2.3) | 1.5 (−0.6; 3.6) |
|  |  | ES (95% CI) | −0.2 (−0.8; 0.5) | 0.2 (−0.5; 0.8) | −0.2 (−0.9; 0.5) | −0.6 (−1.4; 0.2) |
|  | NTG *vs.* CG | MD (95% CI) | 0.3 (−1.3; 1.9) | −1.4 (−3.1; 0.2) | 1.1 (−0.7; 3.0) | 1.4 (−0.6; 3.4) |
|  |  | ES (95% CI) | −0.1 (−0.8; 0.5) | 0.7 (−0.03; 1.4) | −0.5 (−1.2; 0.2) | −0.6 (−1.3; 0.2) |
|  | MTG *vs.* CG | MD (95% CI) | −0.1 (−2.0; 1.8) | −1.1 (−2.7; 0.5) | 0.6 (−1.2; 2.5) | −0.1 (−2.1; 2.0) |
|  |  | ES (95% CI) | 0.0 (−0.6; 0.7) | 0.6 (−0.01; 1.3) | 0.01 (−0.7; 0.7) | 0.04 (−0.7; 0.8) |
| Left lateral excursion | NTG *vs.* MTG | MD (95% CI) | 0.5 (−0.9; 1.9) | −0.3 (−2.3; 1.6) | −0.5 (−2.6; 1.6) | 0.6 (−1.2; 2.4) |
|  |  | ES (95% CI) | −0.2 (−0.9; 0.4) | 0.1 (−0.5; 0.8) | 0.2 (−0.5; 0.9) | −0.4 (−1.2; 0.4) |
|  | NTG *vs.* CG | MD (95% CI) | 0.1 (−1.6; 1.8) | −1.7 (−3.7; 0.3) | −0.1 (−2.2; 1.9) | 0.4 (−2.1; 1.4) |
|  |  | ES (95% CI) | −0.1 (−0.7; 0.6) | 0.7 (−0.01; 1.4) | 0.1 (−0.6; 0.8) | 0.2 (−0.6; 0.9) |
|  | MTG *vs.* CG | MD (95% CI) | −0.4 (−2.1; 1.3) | −1.4 (−3.4; 0.5) | 0.3 (−1.7; 2.4) | −1.0 (−2.7; 0.8) |
|  |  | ES (95% CI) | 0.1 (−0.5; 0.8) | 0.6 (−0.1; 1.3) | −0.1 (−0.8; 0.5) | 0.5 (−0.2; 1.3) |
| Protrusion | NTG *vs.* MTG | MD (95% CI) | −0.3 (−1.4; 0.8) | −0.6 (−1.8; 0.7) | −0.3 (−1.6; 1.0) | −0.7 (−2.1; 0.7) |
|  |  | ES (95% CI) | 0.2 (−0.5; 0.8) | 0.4 (−0.3; 1.0) | 0.2 (−0.4; 0.9) | 0.4 (−0.4; 1.2) |
|  | NTG *vs.* CG | MD (95% CI) | 0.0 (−1.0; 1.0) | 0.7 (−0.5; 2.0) | 0.1 (−1.4; 1.2) | 0.2 (−1.1; 1.6) |
|  |  | ES (95% CI) | 0.0 (−0.6; 0.6) | −0.5 (−1.2; 0.2) | 0.1 (−0.6; 0.8) | −0.1 (−0.9; 0.6) |
|  | MTG *vs.* CG | MD (95% CI) | 0.3 (−0.8; 1.4) | 1.3 (0.1; 2.6)* | 0.2 (−1.1; 1.0) | 0.9 (−0.5; 2.3) |
|  |  | ES (95% CI) | −0.2 (−0.8; 0.4) | −0.8 (−1.5; −0.1)* | −0.1 (−0.8; 0.6) | −0.4 (−1.2; 0.4) |

NTG: Cervical training group; MTG: Manual therapy group; CG: control group; MD: Mean difference; ES: standardized effect sizes. **p* < 0.05.

Supplementary Table 3. Within-group mean differences in Pain intensity, Jaw function, and Oral health-related quality of life.

| Outcomes | Groups  (n) | Baseline  mean (SD) | End of treatment  mean (SD) | one-month  follow-up  mean (SD) | three-months  follow-up  mean (SD) | *Post hoc*  within-group (MD 95% CI) | | | | | |
| --- | --- | --- | --- | --- | --- | --- | --- | --- | --- | --- | --- |
|  |  |  |  |  |  | Baseline  *vs.*  End of treatment  MD (95% CI) | Baseline  *vs.*  One-month follow-up | Baseline  *vs.*  Three-months follow-up | End of treatment *vs.*  One-month follow-up | End of treatment  *vs.*  Three-months follow-up | One-month follow-up  *vs.*  Three-months follow-up |
| Pain | NTG (18) | 7.1 (1.7) | 1.8 (1.9) | 1.5 (1.4) | 1.4 (1.4) | 5.3 (4.1; 6.5)* | 5.6 (4.5; 5.6)* | 5.7 (4.6; 6.7)* | 0.3 (−0.8; 1.4) | 0.4 (−0.7; 1.5) | 0.1 (−0.8; 1.0) |
|  | MTG (18) | 6.5 (2.2) | 2.6 (3) | 2.6 (2.6) | 2.4 (2.3) | 3.9 (2.1; 5.6)* | 3.9 (2.3; 5.5)* | 4.1 (2.6; 5.6)* | 0 (−1.9; 1.9) | 0.2 (−1.6; 2.0) | 0.2 (−1.5; 1.9) |
|  | CG (18) | 7.0 (1.6) | 3.7 (2.4) | 2.9 (2.0) | 2.8 (2.0) | 3.3 (1.9; 4.7)* | 4.1 (2.9; 5.3)* | 4.2 (3; 5.4)* | 0.8 (−0.7; 2.3) | 0.9 (−0.6; 2.4) | 0.1 (−1.2; 1.4) |
| OHRQoL | NTG (18) | 20.1 (5.3) | 7.4 (6.2) | 6.9 (7.5) | 5.5 (9.0) | 12.7 (8.8; 16.6)* | 13.2 (8.8; 17.6)* | 14.6 (9.6; 19.6)* | 0.5 (−4.2; 5.2) | 1.9 (−3.3; 7.1) | 1.4 (−4.2; 7.0) |
|  | MTG (18) | 26.2 (15.2) | 15.9 (10.9) | 15.2 (15.0) | 17.2 (17.0) | 10.3 (1.3; 19.2)* | 11 (0.8; 21.2)* | 9 (1.9; 19.9)* | 0.7 (−8.2; 9.6) | −1.3 (−11; 8.4) | −0.2 (−12.8; 8.8) |
|  | PG (18) | 20.9 (9.1) | 16.6 (11.3) | 14.1 (14.0) | 12.8 (11.8) | 4.3 (−2.6; 11.2) | 6.8 (−1.2; 14.8) | 8.2 (1; 15.2)* | 2.5 (−6.1; 11.1) | 3.8 (−4; 11.6) | 1.3 (−7.5; 10.1) |
| Jaw function | NTG (18) | 17.4 (9.1) | 11.9 (7.6) | 9.4 (8.2) | 7.1 (8.2) | 5.5 (−0.2; 11.2) | 8 (2.1; 13.9)* | 10.3 (4.4; 16.2)* | 2.5 (−2.8; 7.8) | 4.8 (−0.5; 10.1) | 2.3 (−3.2; 7.8) |
|  | MTG (18) | 22.8 (12.5) | 17.6 (12.4) | 16.3 (14.6) | 19.6 (15.4) | 5.2 (−3.2; 13.6) | 6.5 (−2.7; 15.7) | 3.2 (−6.3; 12.7) | 1.3 (−7.9; 10.5) | −2 (−11.5, 7.5) | −3.3 (−13.5; 6.9) |
|  | PG (18) | 22.7 (11.2) | 21.4 (12) | 17.4 (12.2) | 16.1 (12.0) | 1.3 (−6.6; 9.2) | 5.3 (−2.6; 13.2) | 6.6 (−4.2; 14.5) | 4 (−1.5; 12.2) | 5.3 (−2.8; 13.4) | 1.3 (−6.9; 9.5) |

N: number of participants; NTG: Neck motor control training group; MTG: Manual therapy group; PG: Placebo group; OHRQoL: oral healthy related quality of life; SD: standard deviations; MD: Mean difference; CI: confidence interval. *: *p* < 0.05.

Supplementary Table 4. Within-group mean differences in jaw movements.

| Outcomes | Groups  (n) | Baseline  mean (SD) | End of treatment  mean (SD) | One-month  follow-up  mean (SD) | Three-months  follow-up  mean (SD) | *Post hoc*  within-group (MD 95% CI) | | | | | |
| --- | --- | --- | --- | --- | --- | --- | --- | --- | --- | --- | --- |
|  |  |  |  |  |  | Baseline  *vs.*  End of treatment | Baseline  *vs.*  One-month follow-up | Baseline  *vs.*  Three-months follow-up | End of treatment *vs.*  One-month follow-up | End of treatment  *vs.*  Three-months follow-up | One-month follow-up  *vs.* Three-months follow-up |
| Jaw opening | NTG (18) | 31.4 (9.0) | 33.3 (8.9) | 33.7 (11.1) | 35.2 (10.1) | −1.9 (−8; 4.2) | −2.3 (−9.1; 4.5) | −3.8 (−10.3; 2.7) | −0.4 (−7.2; 6.4) | −1.9 (−8.3; 4.5) | −1.5 (−8.7; 5.7) |
|  | MTG (18) | 32.3 (8.9) | 35.7 (8.8) | 34.8 (8.4) | 35.2 (6.9) | −3.4 (−9.4; 2.6) | −2.5 (−8.4; 3.4) | −2.9 (−8.3; 2.5) | 0.9 (−4.9; 6.7) | 0.5 (−4.8; 5.8) | −0.4 (−5.6; 4.8) |
|  | PG (18) | 31.2 (10.5) | 29.9 (9.6) | 33.4 (9.1) | 34 (7.8) | 1.3 (−5.5; 8.1) | −2.2 (−8.6; 4.4) | −2.8 (−9.1; 3.5) | −3.5 (−9.8; 2.8) | −4.1 (−10.0; 1.8) | −0.6 (−6.3; 5.1) |
| Right lateral excursion | NTG (18) | 8.2 (1.9) | 7.8 (2.4) | 9.1 (2.6) | 8.7 (1.7) | 0.4 (−1.1; 1.9) | −0.9 (−2.4; 0.6) | −0.5 (−1.7; 0.7) | −1.3 (−3.0; 0.4) | −0.9 (−2.3; 0.5) | 0.4 (−1.1; 1.9) |
|  | MTG (18) | 7.8 (2.7) | 7.9 (1.5) | 8.6 (1.9) | 8.1 (1.6) | −0.1 (−1.6; 1.4) | −0.8 (−2.4; 0.8) | −0.3 (−1.8; 1.2) | −0.7 (−1.9; 0.4) | −0.2 (−1.2; 0.8) | 0.5 (−0.7; 1.7) |
|  | PG (18) | 7.9 (2.8) | 9.1 (2.1) | 8.3 (1.9) | 8.3 (1.6) | −1.2 (−2.9; 0.5) | −0.4 (−2; 1.2) | −0.4 (−1.9; 1.1) | 0.8 (−0.6; 2.1) | 0.8 (−0.5; 2.1) | 0.0 (−1.2; 1.2) |
| Left lateral excursion | NTG (18) | 8.7 (2.1) | 9.1 (2.9) | 8.7 (1.6) | 9.6 (1.8) | −0.4 (−2.1; 1.3) | 0.0 (−1.3; 1.3) | −0.9 (−2.2; 0.4) | 0.4 (−1.2; 2.0) | −0.5 (−2.1; 1.1) | −0.9 (−2; 0.2) |
|  | MTG (18) | 8.2 (2.0) | 9 (2.2) | 9.7 (2.2) | 9.1 (1.3) | −0.8 (−2.2; 0.6) | −1.5 (−2.9; −0.1)* | −0.9 (−2.0; 0.2) | −0.7 (−2.2; 0.8) | −0.1 (−1.3; 1.1) | 0.6 (−0.6; 1.8) |
|  | PG (18) | 8.6 (3.0) | 10.5 (2.5) | 9.5 (2.5) | 9.8 (2.0) | −1.9 (−3.8; −0.1)* | −0.9 (−2.8; 0.1) | −1.2 (−2.9; 0.5) | 1 (−0.7; 2.7) | 0.7 (−0.8; 2.2) | −0.3 (−1.8; 1.2) |
| Protrusion | NTG (18) | 3.9 (1.5) | 4.7 (1.3) | 4.5 (1.1) | 5 (1.7) | −0.8 (−1.7; 0.1) | −0.6 (1.5; 0.3) | −1.1 (−2.2; −0.1)* | 0.2 (−0.6; 1.0) | −0.3 (−1.3; 0.7) | −0.5 (−1.5; 0.5) |
|  | MTG (18) | 4.2 (1.7) | 5.1 (1.8) | 4.6 (1.9) | 5.5 (2.1) | −0.9 (−2.1; 0.3) | −0.4 (−1.6; 0.8) | −1.3 (−2.6; −0.01)* | 0.5 (−0.7; 1.7) | −0.4 (−1.7; 0.9) | −0.9 (−2.2; 0.4) |
|  | PG (18) | 3.9 (1.6) | 3.8 (1.6) | 4.5 (1.7) | 4.5 (1.0) | 0.1 (−1; 1.2) | −0.6 (−1.7; 0.5) | −0.6 (−1.5; 0.3) | −0.7 (−1.8; 0.4) | −0.7 (−1.6; 0.2) | 0.0 (−0.9; 0.9) |

N: number of participants; NTG: Neck motor control training group; MTG: Manual therapy group; PG: placebo group; SD: standard deviations; MD: Mean difference; CI: confidence interval. *: *p* < 0.05.
